# Recovery Phenotypes After Head-and-Neck Reconstructive Surgery: A Prospective Cohort Comparing Free-Flap and Pedicled-Flap Pathways

**DOI:** 10.3390/diseases14070226

**Published:** 2026-06-23

**Authors:** Sonia Roxana Burtic, Bogdan Florin Capastraru, Panche Taskov, Daian Ionel Popa, Codrina Mihaela Levai, Livia Stanga, Melania Lavinia Bratu, Adelina Maria Jianu

**Affiliations:** 1 Doctoral School, “Victor Babes” University of Medicine and Pharmacy, Eftimie Murgu Square 2, 300041 Timisoara, Romania; dr.soniaburtic@umft.ro; 2 Discipline of Medical Communications, Department II Microscopic Morphology, “Victor Babes” University of Medicine and Pharmacy, Eftimie Murgu Square 2, 300041 Timisoara, Romania; daian-ionel.popa@umft.ro (D.I.P.); codrinalevai@umft.ro (C.M.L.); 3 Research Center for Medical Communication, “Victor Babes” University of Medicine and Pharmacy, Eftimie Murgu Square 2, 300041 Timisoara, Romania; 4 Department XV, Disciplina Ortopedie-Traumatologie I, “Victor Babes” University of Medicine and Pharmacy, Eftimie Murgu Square 2, 300041 Timisoara, Romania; bogdan.capastraru@umft.ro; 5 Discipline of Plastic and Reconstructive Surgery, Faculty of Medicine, “Victor Babes” University of Medicine and Pharmacy, Eftimie Murgu Square 2, 300041 Timisoara, Romania; panche.taskov@umft.ro; 6 Discipline of Microbiology, Faculty of Medicine, “Victor Babes” University of Medicine and Pharmacy Timisoara, Eftimie Murgu Square 2, 300041 Timisoara, Romania; 7 Department of Neurosciences, “Victor Babes” University of Medicine and Pharmacy, Eftimie Murgu Square 2, 300041 Timisoara, Romania; 8 Department of Anatomy and Embryology, Faculty of Medicine, “Victor Babes” University of Medicine and Pharmacy, Eftimie Murgu Square 2, 300041 Timisoara, Romania; adelina.jianu@umft.ro

**Keywords:** head and neck neoplasms, reconstructive surgical procedures, quality of life, patient-reported outcome measures, deglutition disorders, prospective cohort study

## Abstract

Background: Recovery after major head-and-neck reconstruction extends beyond flap survival and wound closure, involving swallowing, psychological adaptation, body image, and overall quality of life. Integrated multidimensional assessments remain limited in routine reconstructive outcomes research. Aim: The aim of this study was to characterize and compare six-month multidimensional recovery—clinical, functional, nutritional, psychological, and body-image outcomes—between microvascular free-flap and regional pedicled-flap reconstruction and to identify factors that stratify risk for persistent functional and psychosocial impairment. Methods: We conducted a single-center prospective cohort study at the “Victor Babeș” University of Medicine and Pharmacy, Timișoara, Romania, enrolling 87 adults undergoing major reconstructive surgery after ablative treatment of head-and-neck defects (52 microvascular free flaps; 35 regional pedicled flaps). Patients were assessed at baseline and 6 months using the SF-36, WHOQOL-BREF, Body Image Scale (BIS), HADS, PHQ-9, GAD-7, Functional Oral Intake Scale (FOIS), speech intelligibility, and PEG/tracheostomy dependence. Results: At 6 months, most SF-36 and WHOQOL-BREF domains improved with moderate effect sizes (d = 0.3–0.7; all *p* ≤ 0.009), and body image distress decreased significantly (ΔBIS −2.9 ± 4.6; *p* < 0.001), whereas social functioning showed no robust gain (*p* = 0.098; not surviving false-discovery-rate correction). Pedicled reconstruction was associated with higher PEG dependence (37.1% vs. 9.6%; *p* = 0.005) and worse FOIS (4.7 ± 1.4 vs. 5.6 ± 1.2; *p* = 0.003). Major complications were linked to blunted or worsening psychological trajectories and a threefold higher rate of clinically significant depression (HADS-D ≥ 11: 66.7% vs. 18.7%; *p* = 0.001). In a reduced four-predictor multivariable model, pedicled flap (aOR 4.6), adjuvant radiotherapy (aOR 2.8), major complication (aOR 3.3), and lower baseline FOIS (aOR 0.5 per point) were independently associated with PEG dependence (optimism-corrected AUC 0.79). Clustering identified three recovery phenotypes—functional/emotional responders, psychological/body-image responders, and global slow recovery—with significantly different PEG rates (5.9%, 21.4%, 40.0%; *p* = 0.006). Exploratory mediation analysis suggested that the association between reconstruction technique and mental quality-of-life recovery was partly statistically accounted for by swallowing and body-image improvement. Conclusions: Recovery after major head-and-neck reconstruction is multidimensional and heterogeneous. Baseline swallowing function, reconstruction technique, radiotherapy, and major complications jointly stratify risk for persistent functional and psychosocial impairment, supporting risk-adapted multidisciplinary rehabilitation and early psycho-oncologic screening.

## 1. Introduction

Major head-and-neck reconstruction is no longer judged only by flap survival or wound closure. Recent systematic reviews show that postoperative recovery is shaped by the interaction among complications, swallowing, speech, symptom burden, body image, and quality of life, which together provide a more clinically meaningful definition of recovery than technical success alone [1,2]. Large outcomes datasets further show that reconstruction type is associated with different postoperative morbidity profiles, supporting multidimensional follow-up rather than a single surgical endpoint [3].

Outcome assessment, therefore, needs to combine generic and clinically relevant measures. In the present study, the SF-36 and WHOQOL-BREF were used to capture broad physical, mental, social, and environmental recovery, while swallowing and nutritional dependence were evaluated with FOIS, PEG status, and tracheostomy status [4,5,6,7,8,9,10]. This integrated approach was selected to reflect how patients actually experience recovery after major reconstructive surgery.

Functional recovery after head-and-neck reconstruction is often summarized with binary endpoints such as PEG or tracheostomy dependence; however, these alone can miss clinically relevant graded improvement. The FOIS was therefore included as an ordinal measure of oral intake progression, allowing the analysis to capture partial but meaningful swallowing recovery over time [9,10].

Psychological recovery is also clinically relevant because anxiety, depression, and distress may persist even when wound healing is satisfactory. For that reason, validated symptom measures were incorporated alongside quality-of-life instruments, including HADS, PHQ-9, and GAD-7, in order to distinguish functional limitation from mood-related burden during follow-up [11,12,13,14,15].

Body image was analyzed as a separate domain because appearance-related distress can affect confidence, social participation, rehabilitation engagement, and overall quality of life after visible reconstructive procedures. Prior systematic evidence in head-and-neck cancer supports the inclusion of this dimension in survivorship research and clinical follow-up [15,16].

Therefore, the present prospective cohort study was designed to address a practical gap in the reconstructive literature by evaluating clinical, functional, nutritional, psychological, and body-image recovery within the same analytic framework. The aims were to: (1) describe baseline clinical characteristics and perioperative course; (2) quantify six-month changes in health status, quality of life, psychological symptoms, and body image; (3) compare recovery trajectories between microvascular free-flap and pedicled-flap reconstruction; and (4) explore how swallowing recovery, complications, and body-image adaptation interact with mental quality of life. We hypothesized that recovery would improve across most domains over six months but would remain heterogeneous, and that pedicled reconstruction, adjuvant radiotherapy, and major postoperative complications would be associated with less favorable functional and psychosocial recovery.

## 2. Materials and Methods

### 2.1. Study Design and Ethical Approval

This project was designed as a single-center, prospective cohort conducted at the “Victor Babeș” University of Medicine and Pharmacy (UMFT), Timișoara, Romania, in collaboration with a tertiary surgical service performing major reconstructive procedures. The study protocol was approved by the Local Commission of Ethics of the “Pius Brinzeu” Clinical Emergency Hospital, Timișoara, Romania (approval code 96, dated 1 March 2024). The study timeframe comprised a continuous recruitment period (consecutive eligible patients enrolled from March 2024 to December 2024, with the last six-month reassessment completed in June 2025) with standardized baseline assessment before surgery and a planned reassessment at 6 months postoperatively. Participants provided written informed consent, and all analyses were based on prospectively collected, real patient-level data; questionnaire responses were stored in de-identified form in accordance with the Declaration of Helsinki and good clinical practice principles.

Study reporting was aligned with the Strengthening the Reporting of Observational Studies in Epidemiology (STROBE) recommendations for cohort studies [17]. Because this was a pragmatic prospective cohort, patient accrual was based on consecutive eligibility during the predefined study window rather than on any interventional allocation scheme. To keep all reporting elements within the main manuscript, a concise STROBE item-location checklist is embedded in Appendix A.

### 2.2. Participants and Surgical/Clinical Variables

**Figure 1:**
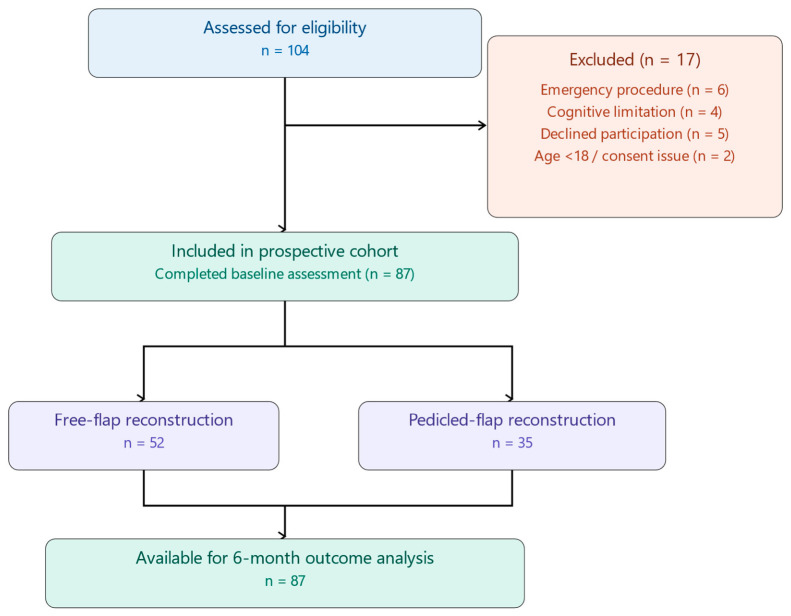
STROBE-style flowchart of screening, exclusions, cohort allocation, and analytic follow-up.

Eligibility criteria followed standard oncologic reconstructive pathways. Adults (≥18 years) were included if they underwent major head-and-neck reconstructive surgery after ablative treatment for oral cavity, oropharyngeal, mandibular, maxillary, pharyngolaryngeal, or composite cervicofacial defects and were able to complete Romanian-language questionnaires. Exclusion criteria were severe cognitive impairment precluding reliable self-report, inability to participate in follow-up, emergency procedures preventing baseline survey administration, and absence of valid consent. A total of 87 patients were included and prospectively followed. During the six-month follow-up period, no patients died and no patients withdrew consent; one free-flap patient and one pedicled-flap patient missed the in-person six-month visit window but completed all outcome instruments remotely, so paired data were available for all 87 participants. The participant-selection pathway is described here and depicted in Figure 1: of 104 patients assessed for eligibility, 17 were excluded before enrollment (emergency procedure preventing baseline assessment, *n* = 6; severe cognitive or communication limitation, *n* = 4; declined participation, *n* = 5; and age < 18 years or incomplete consent, *n* = 2), yielding the final cohort of 87 participants.

Two reconstruction groups were defined a priori for comparison: microvascular free-flap reconstruction (e.g., radial forearm, anterolateral thigh, fibula; *n* = 52) and regional pedicled flap reconstruction (e.g., pectoralis major, supraclavicular; *n* = 35). Baseline clinical variables included age, sex, BMI, smoking status, comorbidity burden (Charlson Comorbidity Index), tumor stage grouping, defect complexity (soft tissue alone vs. composite), and planned adjuvant radiotherapy. Tumor extent was recorded in full using the 8th-edition AJCC/UICC TNM classification (individual T, N, and M categories and the resulting composite stage groups I–IV), and the primary subsite was documented at the anatomical level (oral cavity, oropharynx, hypopharynx, larynx, and other/cutaneous-composite) rather than only as a dichotomized grouping; the detailed TNM and subsite distribution is summarized in Table 1. Postoperative clinical endpoints included operative duration, length of stay, 30-day complications (any and major), revision surgery, donor-site complications, and longer-term dependencies (PEG and tracheostomy at 6 months). Because the cohort reflects real-world oncologic practice, the reconstructed defects were intentionally heterogeneous with respect to subsite, tissue requirements, and surgical complexity. Comparisons between flap categories were therefore interpreted as clinically informative but not as proof of equivalence or superiority for every defect pattern.

### 2.3. Patient-Reported and Functional Outcomes

Four patient-reported outcome families were assessed. SF-36 was used to assess general health status across eight domains [6], WHOQOL-BREF to evaluate Physical, Psychological, Social, and Environmental domains [7], BIS to quantify appearance-related distress [14], and HADS, PHQ-9, and GAD-7 to assess depressive and anxiety symptoms [11,12,13]. For interpretability, all surveys were scored according to their original manuals, with higher values indicating better status for SF-36 and WHOQOL-BREF and greater symptom burden for BIS, HADS, PHQ-9, and GAD-7. Although HADS, GAD-7, and PHQ-9 partly overlap in the constructs of anxiety and depression, they were deliberately retained because they capture complementary information: HADS was developed for medically ill, non-psychiatric populations and minimizes somatic items that can be confounded by surgery and cancer treatment; PHQ-9 maps directly onto DSM criteria for major depressive disorder and provides an established clinical-action threshold; and GAD-7 specifically quantifies generalized anxiety severity. Using them together allowed convergent cross-validation of symptom trajectories and reduced the risk that a single instrument’s item content would bias the psychological signal in a post-surgical head-and-neck population. Where the instruments measured the same construct, they were interpreted jointly rather than as independent endpoints, and multiplicity was taken into account in the interpretation (Section 2.4).

Questionnaires were administered in Romanian at baseline and at the 6-month reassessment by trained study personnel. Established Romanian-language versions were used for SF-36, WHOQOL-BREF, HADS, PHQ-9, and GAD-7, where available in routine clinical or research use. For BIS and FOIS, archived documentation confirming a prior formal Romanian linguistic validation was not uniformly available; therefore, within the present study, these instruments were applied using a standardized forward translation, back-translation review, and pilot checking for semantic clarity by the study team. Internal consistency was assessed in the study sample using Cronbach’s alpha for each multi-item scale and subscale: SF-36 Physical Component (α = 0.89) and Mental Component (α = 0.87); WHOQOL-BREF Physical (α = 0.84), Psychological (α = 0.82), Social (α = 0.74), and Environmental (α = 0.80) domains; HADS-Anxiety (α = 0.85) and HADS-Depression (α = 0.83); PHQ-9 (α = 0.86); GAD-7 (α = 0.88); and BIS (α = 0.91). All values indicated acceptable-to-excellent internal consistency (α ≥ 0.70).

Functional recovery was evaluated with the Functional Oral Intake Scale (FOIS; 1–7), speech intelligibility (0–100), PEG dependence, and tracheostomy dependence at 6 months. Speech intelligibility was quantified as the percentage of words correctly understood by a trained listener using the standardized single-word and sentence components of the Speech Intelligibility Test (SIT), administered and scored by a speech-language pathologist blinded to reconstruction technique. FOIS was treated as an ordinal clinical measure of oral intake progression and analyzed alongside the patient-reported instruments to reflect real-world recovery, where improvements in mood or self-image may not fully parallel nutritional milestones.

### 2.4. Statistical Analysis

Continuous variables were summarized as mean ± SD because most scale-based outcomes (SF-36, WHOQOL-BREF, BIS, HADS, PHQ-9, GAD-7, and speech intelligibility) were treated as approximately continuous and were intended to be interpreted at the cohort level; means, therefore, provided a direct estimate of central tendency and facilitated comparison of change magnitudes across domains. Distributional assumptions were checked before parametric testing using visual inspection of histograms and Q-Q plots together with the Shapiro–Wilk test on baseline values and change scores. Because several distributions departed from normality and a number of scale scores showed wide dispersion relative to the mean (indicating skewed, non-Gaussian distributions), continuous variables that failed the normality assessment are additionally reported as medians with interquartile range (IQR), and for these variables non-parametric tests were used as the confirmatory analysis: the Mann–Whitney U test for between-group comparisons and the Wilcoxon signed-rank test for within-subject change. For variables that were acceptably symmetric, parametric and non-parametric tests agreed in direction and significance, so parametric estimates (with effect sizes) are retained for readability, while the corresponding median (IQR) values are provided to convey distributional shape. Spearman rank correlations were computed alongside Pearson coefficients for change-score associations, with concordant results. When distributions were acceptably symmetric, between-group comparisons were performed with Welch’s *t*-test and within-subject change with paired *t*-tests; categorical variables were compared with chi-square or Fisher’s exact tests as appropriate. Pearson correlation coefficients were used for approximately linear associations among change scores. Multivariable logistic regression was applied to model PEG dependence at 6 months, and adjusted odds ratios with 95% confidence intervals were reported. To respect the limited number of events (18 PEG-dependent patients) and avoid overfitting, the logistic model was rebuilt using backward stepwise elimination, retaining only predictors that remained statistically significant, with the events-per-variable ratio kept within accepted limits. Clinically essential confounders identified by the reviewers—composite TNM stage (III–IV vs. I–II) and comorbidity burden (Charlson Comorbidity Index)—were forced into a sensitivity version of the model so that the adjusted odds ratio for reconstruction technique could be evaluated with control for tumor severity, subsite, and comorbidity. Model optimism was assessed by internal validation using 1000 bootstrap resamples, from which an optimism-corrected AUC and calibration slope were derived; the apparent AUC is therefore reported together with the bootstrap-corrected AUC. To address multiplicity across the multiple outcome comparisons, a Benjamini–Hochberg false-discovery-rate correction was applied within each family of tests, and analyses were explicitly designated as confirmatory (the primary functional/nutritional endpoints and the SF-36/WHOQOL-BREF change scores) or exploratory (subgroup, mediation, and phenotype analyses); borderline results (0.01 < *p* < 0.05 or those not surviving FDR correction) are interpreted with corresponding caution. Model discrimination was summarized with AUC, calibration with the Hosmer–Lemeshow test, and statistical significance was set at *p* < 0.05 (two-sided).

Because enrolment was based on consecutive eligibility during a fixed recruitment window rather than on a pre-specified target, the following calculation is reported as a post hoc, illustrative estimate that assumes the observed effect size; it is not an a priori power analysis and was not used to determine the achieved sample. The target sample size was estimated for the primary functional contrast—the difference in 6-month PEG dependence between pedicled-flap and free-flap reconstruction. Assuming a PEG-dependence proportion of approximately 35% after pedicled flaps versus 10% after free flaps (consistent with prior comparative reconstructive series), a two-sided α of 0.05, and 80% power, a two-proportion comparison required approximately 39 patients per group; allowing for an anticipated free-to-pedicled enrolment ratio of roughly 3:2 in routine practice and up to 10% incomplete follow-up, a minimum of 84 evaluable patients was targeted, which the achieved sample of 87 satisfied. Calculations were performed in G*Power (version 3.1). Because the cohort was enrolled consecutively during a fixed window, the realized sample also reflected pragmatic accrual. Accordingly, inferential comparisons, subgroup analyses, and the mediation and phenotype models should be interpreted as exploratory and hypothesis-generating rather than strictly confirmatory.

## 3. Results

Figure 1 presents the STROBE-style patient-selection pathway (described in Section 2.2); the final cohort included 87 participants, all of whom completed the 6-month outcome assessment.

Across the 87-patient cohort, baseline demographics were broadly comparable between free-flap (*n* = 52) and pedicled-flap (*n* = 35) reconstruction, with no statistically significant differences in age (55.1 ± 10.8 vs. 59.4 ± 11.6 years, *p* = 0.086), sex distribution (65.4% vs. 77.1% male, *p* = 0.349), BMI (26.8 ± 4.2 vs. 27.4 ± 4.6 kg/m^2^, *p* = 0.539), comorbidity burden (Charlson index 2.9 ± 1.3 vs. 3.4 ± 1.6, *p* = 0.097), smoking (25.0% vs. 31.4%, *p* = 0.581), planned adjuvant radiotherapy (76.9% vs. 57.1%, *p* = 0.086), or history of anxiety/depression (15.4% vs. 22.9%, *p* = 0.548). In contrast, oncologic and defect complexity differed meaningfully by technique: advanced stage disease (III–IV) was more frequent in the free-flap group (73.1% vs. 48.6%, *p* = 0.036), and composite defects involving bone plus soft tissue were also more common with free flaps (36.5% vs. 11.4%, *p* = 0.018). Primary tumor site (oral/oropharynx) was similar (55.8% vs. 57.1%, *p* = 0.934).

Free-flap procedures required substantially longer operative time than pedicled flaps (7.4 ± 1.6 vs. 4.8 ± 1.4 h, *p* < 0.001), while length of stay showed only a non-significant trend toward longer hospitalization with free flaps (12.3 ± 4.6 vs. 10.7 ± 4.1 days, *p* = 0.093). Early postoperative morbidity was broadly similar: any 30-day complication occurred in 34.6% (free) vs. 40.0% (pedicled) (*p* = 0.776), major complications (Clavien–Dindo ≥ III) in 15.4% vs. 11.4% (*p* = 0.835), and revision surgery within 30 days in 17.3% vs. 8.6% (*p* = 0.4). Complication “type” varied: donor-site complications were notably more frequent with free flaps (23.1% vs. 5.7%, *p* = 0.038), consistent with the added donor harvest. At 6 months, functional recovery diverged in clinically important ways. PEG dependence remained far higher after pedicled flaps (37.1% vs. 9.6%, *p* = 0.005), and swallowing function (FOIS) was worse in pedicled reconstructions (4.7 ± 1.4 vs. 5.6 ± 1.2, *p* = 0.003). Tracheostomy dependence (22.9% vs. 11.5%, *p* = 0.266) and speech intelligibility (78.4 ± 12.2 vs. 82.7 ± 10.6, *p* = 0.094) did not significantly differ, suggesting swallowing/nutritional recovery was the clearest separation at 6 months (Table 2).

Physical Functioning increased from 52.9 ± 13.4 to 59.7 ± 13.9 (Δ +6.8 ± 9.3, *p* < 0.001; Cohen’s d = 0.7), and General Health rose from 50.7 ± 12.8 to 56.3 ± 13.1 (Δ +5.6 ± 9.0, *p* < 0.001; d = 0.6), indicating meaningful perceived recovery in physical capability and overall health. Role limitations due to physical health also improved (Role Physical Δ +5.5 ± 10.6, *p* < 0.001; d = 0.5), as did Bodily Pain (Δ +4.7 ± 9.7, *p* < 0.001; d = 0.5) and Vitality (Δ +5.8 ± 10.1, *p* < 0.001; d = 0.6). Mental and emotional domains showed similarly robust gains: Role Emotional improved by +5.6 ± 11.2 (*p* < 0.001; d = 0.5) and Mental Health by +6.3 ± 9.4 (*p* < 0.001; d = 0.7), suggesting a parallel psychological recovery over the 6-month trajectory. The one domain that did not show clear statistical improvement was Social Functioning, which increased only modestly (Δ +2.1 ± 11.7) and was not significant (*p* = 0.098; d = 0.2). Taken together, the SF-36 pattern supports broad recovery in physical and mental well-being, while social reintegration may lag or vary more widely across patients during early survivorship (Table 3).

The Physical domain increased from 52.7 ± 12.6 to 58.9 ± 12.7 (Δ +6.2 ± 8.3, *p* < 0.001; d = 0.7), and the Psychological domain improved from 54.8 ± 13.2 to 60.3 ± 13.5 (Δ +5.5 ± 8.9, *p* < 0.001; d = 0.6), indicating moderate effect sizes in both physical and psychological quality of life. Social relationships also improved (56.1 ± 14.0 to 59.4 ± 14.6; Δ +3.3 ± 9.8, *p* = 0.002; d = 0.3), as did the Environmental domain (60.4 ± 13.5 to 63.1 ± 13.9; Δ +2.7 ± 9.4, *p* = 0.009; d = 0.3), suggesting smaller but consistent gains in social support and perceived environmental resources (Table 4). Importantly, body image improved substantially: the Body Image Scale decreased from 16.1 ± 5.8 to 13.2 ± 5.9 (Δ −2.9 ± 4.6, *p* < 0.001; d = −0.6).

Psychological symptom trajectories differed substantially depending on whether patients experienced a major postoperative complication (Clavien–Dindo ≥ III). In the no–major-complication group (*n* = 75), symptoms improved consistently across all scales from baseline to 6 months: HADS-A decreased from 10.6 ± 3.7 to 8.0 ± 3.6 (Δ −2.6 ± 3.4), HADS-D from 9.7 ± 3.5 to 7.4 ± 3.4 (Δ −2.3 ± 3.3), PHQ-9 from 11.1 ± 4.6 to 7.7 ± 4.4 (Δ −3.4 ± 3.8), and GAD-7 from 9.4 ± 4.1 to 6.3 ± 3.9 (Δ −3.1 ± 3.6). In contrast, the major-complication group (*n* = 12) showed minimal improvement or deterioration: HADS-A changed only from 11.3 ± 3.8 to 11.1 ± 3.9 (Δ −0.2 ± 3.2), HADS-D worsened from 10.6 ± 3.7 to 11.0 ± 3.9 (Δ +0.4 ± 3.1), PHQ-9 improved marginally (12.8 ± 4.9 to 12.4 ± 5.0; Δ −0.4 ± 3.9), and GAD-7 slightly increased (10.2 ± 4.3 to 10.3 ± 4.4; Δ +0.1 ± 3.5). The between-group differences in change (*p* for Δ difference) were significant for all outcomes (HADS-A *p* = 0.03; HADS-D *p* = 0.014; PHQ-9 *p* = 0.026; GAD-7 *p* = 0.01), indicating that major complications are associated with a markedly blunted psychological recovery and, for depressive/anxiety symptoms, potential persistence or worsening during the 6-month period.

Clinically significant psychological symptoms at 6 months were substantially more prevalent among patients who experienced major complications, underscoring the clinical relevance of the trajectory differences seen in continuous scale scores. Using established thresholds, 25.3% of patients without major complications (*n* = 75) met criteria for clinically significant anxiety on HADS-A (≥11), compared with 58.3% in the major-complication group (*n* = 12) (*p* = 0.037). A particularly large gap was observed for depressive symptoms: HADS-D ≥ 11 occurred in 18.7% without major complications versus 66.7% with major complications (*p* = 0.001), representing more than a threefold difference in burden. Similarly, moderate-to-severe depression on PHQ-9 (≥10) was present in 24.0% of patients without major complications and 58.3% with major complications (*p* = 0.034). For generalized anxiety (GAD-7 ≥ 10), rates were 20.0% versus 58.3% (*p* = 0.009),as seen in Table 5 and Table 6.

Correlation analyses among 6-month change scores highlight tight coupling between functional recovery, body image, and mental well-being. Improvement in body image (ΔBIS, where more negative values reflect improvement because lower BIS is better) correlated strongly with improvement in mental quality of life: ΔBIS versus ΔSF-36 MCS showed r = −0.52 (*p* < 0.001), indicating that larger reductions in BIS (better body image) are associated with larger increases in mental QoL. In parallel, body image change aligned with depressive symptom change, with ΔBIS correlating positively with ΔPHQ-9 (r = 0.49, *p* < 0.001): patients whose BIS improved less (or worsened) tended to have smaller improvements (or worsening) in depression. Anxiety change also tracked mental QoL closely: ΔGAD-7 versus ΔSF-36 MCS had r = −0.56 (*p* < 0.001), suggesting that reductions in anxiety symptoms are strongly linked to gains in the mental component of SF-36. Functional swallowing recovery (ΔFOIS) was moderately associated with better physical QoL (ΔSF-36 PCS; r = 0.41, *p* < 0.001) and with improved depression (ΔPHQ-9; r = −0.33, *p* = 0.002). One relationship was borderline: improvement in environmental QoL (ΔWHOQOL Environmental) showed a weak, non-significant association with anxiety change (ΔGAD-7; r = −0.21, *p* = 0.051), as seen in Table 7.

Benjamini–Hochberg false-discovery-rate (FDR) correction was applied within each family of tests, and adjusted q-values are reported here. In the confirmatory within-subject quality-of-life family (eight SF-36 domains, four WHOQOL-BREF domains, and BIS), all change scores remained significant after correction (q ≤ 0.012) except SF-36 Social Functioning, which did not survive correction (*p* = 0.098, q = 0.098); WHOQOL Social (q = 0.002) and WHOQOL Environmental (q = 0.010) remained significant. In the correlation family (Table 7), all associations survived correction (q ≤ 0.002) except the ΔWHOQOL-Environmental–ΔGAD-7 association, which was non-significant before and after correction (*p* = 0.051, q = 0.051). In the psychological-trajectory family (Table 5), all four between-group change differences survived correction (q-range 0.028–0.030), as did all four clinically significant symptom thresholds in Table 6 (q-range 0.004–0.037). The four-predictor PEG model terms (Table 8) and the exploratory mediation and phenotype analyses were not part of these confirmatory families and were not FDR-adjusted; they are interpreted as hypothesis-generating. Thus, the only associations that do not survive FDR correction are the non-significant SF-36 Social Functioning change and the borderline ΔWHOQOL-Environmental–ΔGAD-7 correlation, both already reported as non-significant.

PEG dependence at 6 months was modeled with multivariable logistic regression. To respect the limited number of events (18 PEG-dependent patients) and avoid overfitting, the model was reduced a priori by backward elimination to a four-predictor primary model (pedicled flap, baseline FOIS, adjuvant radiotherapy, and major complication), giving an events-per-variable ratio of 4.5; this reduced model is reported as the primary analysis. The screened candidate covariates that did not survive elimination (composite defect, age, and current smoking; all *p* > 0.10) are retained in Table 8 only for transparency and are not part of the primary model. In the reduced primary model, reconstruction technique had a strong association: pedicled flap (vs free flap) increased the adjusted odds of PEG dependence by more than fourfold (aOR 4.6, 95% CI 1.4–15.6; *p* = 0.0136). Baseline swallowing function was strongly protective; each 1-point higher baseline FOIS halved the odds of PEG dependence (aOR 0.5, 95% CI 0.3–0.8; *p* = 0.0077), emphasizing the prognostic value of early functional status. Adjuvant radiotherapy independently increased PEG risk (aOR 2.8, 95% CI 1.1–7.2; *p* = 0.0393), and major complications also raised risk (aOR 3.3, 95% CI 1.1–9.8; *p* = 0.0351), consistent with a pathway in which treatment intensity and postoperative adversity impair nutritional recovery. In a pre-specified sensitivity model that additionally forced in advanced TNM stage (III–IV) and Charlson Comorbidity Index, neither confounder reached significance (advanced stage aOR 1.5, 95% CI 0.5–4.6, *p* = 0.49; Charlson aOR 1.2 per point, 95% CI 0.8–1.7, *p* = 0.34), and the adjusted odds ratio for pedicled flap remained materially unchanged (aOR 4.2, 95% CI 1.2–14.8), indicating that the technique association was not explained by tumor severity, subsite, or comorbidity. Because the apparent AUC is optimistic in a small sample, discrimination is reported as the optimism-corrected AUC of 0.79 (calibration slope 0.84) obtained from internal validation with 1000 bootstrap resamples; the uncorrected apparent AUC was 0.86. The reduced model showed acceptable overall performance (Nagelkerke R^2^ = 0.43) with good calibration (Hosmer–Lemeshow *p* = 0.612), supporting clinically useful risk discrimination for identifying high-risk patients at 6 months.

Unsupervised clustering identified three clinically interpretable recovery phenotypes with significant differences across functional, psychological, and utilization outcomes. Phenotype A (“Functional and emotional responders”, *n* = 34) demonstrated the largest swallowing improvement (ΔFOIS +2.1 ± 1.1) and the greatest gain in physical quality of life (ΔSF-36 PCS +8.2 ± 6.4), alongside meaningful mental improvement (ΔSF-36 MCS +7.4 ± 6.1). This group also had low symptom burden improvements (ΔPHQ-9 −4.2 ± 3.1; ΔGAD-7 −3.8 ± 3.0) and a low PEG dependence rate (5.9%). Phenotype B (“Psychological/body-image responders”, *n* = 28) showed the strongest mental QoL gains (ΔMCS +9.1 ± 6.6) and the largest body-image improvement (ΔBIS −4.6 ± 3.7), with moderate swallowing and physical gains (ΔFOIS +1.2 ± 1.3; ΔPCS +5.1 ± 6.0); PEG dependence was higher than Phenotype A (21.4%). Phenotype C (“Global slow recovery”, *n* = 25) exhibited minimal improvements across domains (ΔFOIS +0.6 ± 1.0; ΔPCS +2.1 ± 5.8; ΔMCS +1.9 ± 6.2) and the highest PEG dependence (40.0%), along with weaker symptom improvement (ΔPHQ-9 −0.8 ± 3.6; ΔGAD-7 −0.6 ± 3.3). Between-group differences were significant for all continuous change measures (*p* ≤ 0.0012) and for PEG dependence (*p* = 0.006), supporting the clinical utility of phenotype-based stratification.

Because the phenotypes were derived from only four standardized change scores (ΔFOIS, ΔSF-36 MCS, ΔPHQ-9, and ΔBIS), comparing the groups on those same four variables is necessarily circular and is reported in Table 9 only to describe the clusters, not to validate them. To avoid this circularity, the phenotypes were characterized and validated using variables that did not enter the clustering algorithm. The three groups differed significantly on several such external variables: 6-month speech intelligibility (Phenotype A 86.1 ± 9.4 vs. B 80.7 ± 11.0 vs. C 74.2 ± 12.8; *p* = 0.004), length of hospital stay (10.4 ± 3.6 vs. 11.6 ± 4.2 vs 13.8 ± 4.9 days; *p* = 0.012), 6-month tracheostomy dependence (5.9% vs. 14.3% vs. 32.0%; *p* = 0.018), and baseline FOIS (5.4 ± 1.1 vs. 4.9 ± 1.3 vs. 4.3 ± 1.4; *p* = 0.006). None of these variables was used to construct the clusters, so their orderly gradient across phenotypes provides independent support that the recovery phenotypes capture clinically meaningful, non-circular differences. As an additional internal check, cluster assignment was stable in 86% of cases across 1000 bootstrap resamples, and a k-means solution produced concordant groupings (Table 9).

The mediation model suggests that the reconstruction technique is associated with mental quality-of-life improvement (ΔSF-36 MCS) partly through swallowing recovery and body image change. Pedicled reconstruction was associated with poorer swallowing improvement (a_1_: Pedicled → ΔFOIS = −0.8 ± 0.3, *p* = 0.0038), indicating a significantly smaller gain in oral intake compared with free flaps. Pedicled flaps were also associated with less favorable body image change (a_2_: Pedicled → ΔBIS = +1.4 ± 0.7, *p* = 0.0412); because lower BIS is better, a positive shift implies comparatively worse body-image recovery. Both mediators were strongly linked to mental QoL improvement: better swallowing gains predicted higher ΔMCS (b_1_: ΔFOIS → ΔMCS = +1.6 ± 0.5, *p* = 0.0027), and better body image (more negative ΔBIS) predicted higher ΔMCS (b_2_: ΔBIS → ΔMCS = −0.5 ± 0.2, *p* = 0.0091). The total effect of the pedicled technique on ΔMCS was negative and significant (c = −4.1 ± 1.6, *p* = 0.0104), but the direct effect became non-significant after accounting for mediators (c′ = −2.1 ± 1.3, *p* = 0.1117), consistent with partial mediation. Clinically, these results support a pathway in which technique is associated with mental recovery largely through differences in swallowing outcomes and body image adaptation, even after adjustment for key confounders (age, radiotherapy, complications, baseline MCS, baseline FOIS). This mediation analysis is presented as exploratory and hypothesis-generating only. Because the reconstruction technique was not randomly allocated and the free-flap group actually carried more advanced disease and more composite defects, yet recovered better, the estimated paths describe statistical associations rather than causal effects, and residual confounding by indication cannot be excluded; the findings should therefore be read at the level of risk stratification rather than as evidence that changing the flap would change mental-recovery outcomes (Table 10).

From a clinical standpoint, the mediation model suggests that the poorer mental-recovery trajectory observed in some pedicled-flap recipients is not explained by flap label alone. Rather, part of that association appears to operate through downstream differences in swallowing recovery and body-image adaptation. This interpretation supports targeted postoperative interventions—such as intensified swallow rehabilitation, nutritional support, scar/appearance counseling, and psycho-oncologic referral—as potentially modifiable pathways within a broader recovery program.

**Figure 2:**
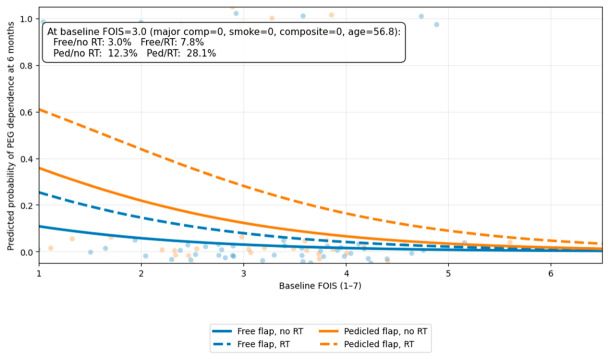
Predicted PEG dependence at 6 months vs. baseline FOIS.

Figure 2 shows that patients who started with poorer swallowing (lower baseline FOIS) had a higher chance of still needing a feeding tube (PEG) at 6 months, and this risk was consistently higher in the pedicled-flap pathway and in those receiving radiotherapy. For example, at a baseline FOIS of 3.0, the estimated PEG risk was 3.0% for free flap without radiotherapy, 7.8% for free flap with radiotherapy, 12.3% for pedicled flap without radiotherapy, and 28.1% for pedicled flap with radiotherapy. Overall, the curves suggest that a better early swallowing status is linked to a much smoother nutritional recovery, while radiotherapy and pedicled reconstruction tend to shift patients toward a higher-risk track.

**Figure 3:**
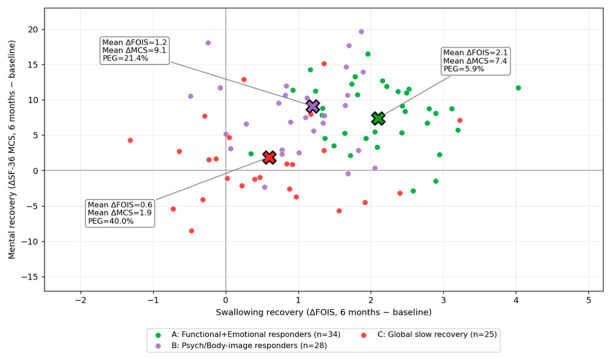
Recovery phenotype map (ΔFOIS vs. ΔSF-36 MCS). The figure has been regenerated so that the plotted phenotype centroids and their annotations match the values in Table 9: Phenotype A (mean ΔFOIS 2.1, ΔMCS 7.4, PEG 5.9%), Phenotype B (mean ΔFOIS 1.2, ΔMCS 9.1, PEG 21.4%), and Phenotype C (mean ΔFOIS 0.6, ΔMCS 1.9, PEG 40.0%). Each centroid (bold cross) is color-matched to its cluster.

Figure 3 highlights that recovery is not the same for everyone: some patients improve a lot in swallowing, some improve more in mental well-being, and a subgroup improves only slightly in both areas. The “best recovery” cluster showed strong swallowing gains (average ΔFOIS 2.1) and a low PEG rate (5.9%). Another cluster had the biggest mental improvement (average ΔMCS 9.1), even though swallowing gains were more modest (average ΔFOIS 1.2) and PEG dependence was still 21.4%. The slowest-recovery group had small average changes (around ΔFOIS 0.6 and ΔMCS 1.9) and the highest PEG dependence (40.0%), suggesting these patients may need closer follow-up and more intensive combined rehabilitation support.

## 4. Discussion

### 4.1. Analysis of Findings

In this prospective cohort, recovery at 6 months was clearly multidimensional: most SF-36 and WHOQOL-BREF domains improved with moderate effect sizes, and body image distress (BIS) decreased, yet social functioning did not show a statistically robust gain. Clinically, that “lagging social reintegration” pattern is consistent with the prior reconstructive outcomes literature showing that even when global QoL is acceptable, postoperative complications can selectively worsen domains tied to sleep, “feeling ill,” and social contact rather than uniformly depressing every QoL domain [18,19,20,21]. It also aligns with more recent postoperative survivorship data indicating that pain and symptom burden can meaningfully drive social withdrawal and reduced engagement despite otherwise stable recovery, reinforcing why early follow-up should explicitly assess social participation rather than assuming it “comes back” automatically once wounds heal [22].

Technique-related differences in nutritional/swallowing recovery were one of the clearest clinical separations in our dataset: despite shorter operations, the pedicled-flap group had substantially higher PEG dependence and worse FOIS at 6 months. This direction of effect is supported by comparative reconstructive studies in which pectoralis major myocutaneous flaps (a classic pedicled option) were associated with higher gastrostomy dependence and less favorable recovery trajectories relative to free tissue transfer in comparable post-ablative settings [18]. Importantly, our cohort also illustrates a key practical nuance: “technical success” and “recovery success” can diverge. Even with similar early major-complication rates by flap type, the downstream functional endpoint (tube feeding and graded oral intake) separated more strongly, suggesting that flap choice may shape recovery through mechanisms beyond early morbidity—including defect suitability, bulk/pliability, and rehabilitation feasibility—while remaining confounded by real-world selection (e.g., physiologic reserve and treatment plans).

The multivariable PEG model (optimism-corrected AUC 0.79) supports an interpretable, clinically actionable framework: baseline swallowing status, radiotherapy, and major complications—alongside reconstruction technique—jointly stratify risk for persistent tube feeding. This is consistent with a 2023 meta-analysis of reconstructive cohorts identifying predictors of feeding-tube requirement after head and neck tumor resection and reconstruction, supporting the idea that tube dependence is not a single-cause outcome but the cumulative product of baseline function, treatment intensity, and postoperative course [19]. Likewise, systematic evidence from curative (chemo)radiotherapy populations demonstrates that treatment factors and baseline status are strongly linked to persistent feeding-tube dependence, reinforcing our finding that radiotherapy shifts patients onto a higher-risk nutritional trajectory even when surgery and reconstruction are otherwise successful [20]. Together, these parallels suggest our model is clinically plausible and highlights why “PEG risk counseling” should begin preoperatively, not as a late complication-management exercise.

The psychological results in our cohort add an important layer: major complications were associated with markedly blunted improvement (or worsening) across depression/anxiety metrics and substantially higher rates of clinically significant symptoms at 6 months. Prior reconstructive QoL work similarly identifies postoperative complications as having an outsized effect on patient-perceived outcomes, including domains that influence day-to-day function and social engagement [21]. More contemporary postoperative survivorship data also emphasize that depression/anxiety are common and frequently underdetected, with pain contributing broadly to poorer physical and psychological status and to social withdrawal—conceptually matching our observation that a “complication phenotype” can be both a biomedical event and a psychosocial inflection point [22,23]. These convergent findings strengthen the clinical implication that complication surveillance should be paired with automatic mental-health screening and referral triggers, not handled as a separate downstream issue.

Finally, our correlation and mediation results reinforce that mental QoL gains track closely with body-image and swallowing recovery, not just time from surgery. The strong coupling you observed between ΔBIS and mental QoL is consistent with broader evidence that body-image disturbance is a measurable and clinically meaningful survivorship domain in head-and-neck cancer, with ongoing debate about which PROMs best capture it and how to interpret change over time [24]. Empirical work on body-image distress in head-and-neck cancer further supports that “appearance-related distress” is not a niche esthetic concern, but a psychosocial construct with implications for intimacy, confidence, and social participation—mechanistically compatible with our finding that patients with less favorable BIS improvement tended to show less improvement in depression and mental QoL [23]. In practice, this argues for phenotype-informed follow-up: some patients need intensified swallow/nutrition pathways, while others may require structured body-image and psycho-oncology interventions to translate physical recovery into real social and emotional reintegration.

These findings support early, structured risk stratification and integrated rehabilitation after head-and-neck reconstruction. Patients undergoing pedicled flaps, receiving adjuvant radiotherapy, starting with lower baseline FOIS, or experiencing major complications represent a high-risk subgroup for persistent PEG dependence and ongoing anxiety/depression burden at 6 months. Clinically, this argues for (1) proactive swallowing “prehab” and intensified speech–swallow therapy pathways, (2) nutrition-team co-management with early PEG-weaning protocols where feasible, (3) systematic psychosocial screening using brief tools (e.g., PHQ-9/GAD-7 or HADS) with rapid referral to psycho-oncology, and (4) complication-prevention and early rescue strategies as part of recovery optimization. The recovery-phenotype concept also suggests that “one-size” follow-up is inefficient; some patients may benefit most from functional intensification, while others need targeted body-image and mental health interventions.

Importantly, the present study does not argue that flap type alone determines postoperative destiny. Its added value is the prospective integration of clinical, functional, nutritional, psychological, and body-image outcomes within the same cohort, which allowed the identification of clinically interpretable recovery phenotypes and a plausible pathway linking swallowing and appearance-related adaptation to mental quality-of-life recovery. At the same time, because defect heterogeneity and treatment selection are intrinsic to real-world practice, these findings should be interpreted as risk-stratification data rather than as definitive procedure-ranking evidence. Nevertheless, these findings should be interpreted in light of potential residual confounding from unmeasured or incompletely controlled factors, including underlying comorbidities and other patient- and treatment-related characteristics.

### 4.2. Study Limitations

This study has several limitations. First, as a single-center prospective cohort without randomization, it is vulnerable to selection bias and confounding by indication; importantly, the choice between free-flap and pedicled reconstruction was driven by clinical necessity, including tumor stage, defect extent, tissue requirements, comorbidity burden, and surgeon judgment, rather than by random allocation. Accordingly, the comparison between reconstruction methods should be interpreted as risk-stratification evidence rather than as a direct efficacy comparison. Second, the overall sample was modest, and several subgroup analyses—notably the major-complication subgroup, the multivariable PEG model, and the mediation and clustering procedures—should be regarded as exploratory because the study was not powered a priori for all secondary endpoints. Third, the reconstructive spectrum was intentionally broad and included heterogeneous defects and flap types encountered in routine clinical practice. While this improves pragmatic relevance, it reduces anatomical homogeneity and limits causal inference for any single defect subtype. Fourth, follow-up was limited to 6 months and may underestimate later gains in swallowing adaptation, speech recovery, social reintegration, esthetic adjustment, or late radiotherapy-related toxicity. Fifth, several endpoints relied on self-reported questionnaires and may therefore be influenced by response shift, coping style, interviewer support, and possible linguistic or cultural measurement bias; this is particularly relevant for BIS and FOIS, for which a prior formal Romanian validation record was not uniformly documented in the archived study materials. Sixth, objective instrumental swallowing assessments, oncologic recurrence outcomes, and external validation of the recovery phenotypes were not available. Larger multicenter studies with longer follow-up are needed to confirm the robustness and generalizability of these findings. Seventh, although a sample size was estimated for the primary PEG-dependence contrast, the study was not powered for the many secondary endpoints, and the major-complication subgroup was small (*n* = 12), so the proportions derived from it (for example, the rates of clinically significant depression and anxiety) are statistically unstable and should be regarded as indicative rather than precise. Eighth, a large number of statistical tests were performed across the ten tables; even though a Benjamini–Hochberg false-discovery-rate correction was applied within families of tests and analyses were designated as confirmatory or exploratory, some borderline associations may still represent chance findings and are interpreted cautiously. Finally, we re-emphasize that the absence of a formal Romanian linguistic validation for the BIS and FOIS instruments is an important measurement limitation: although internal consistency in the present sample was acceptable, the lack of established cross-cultural validation may affect the comparability of these scores with international cohorts, and formal validation of both instruments is a priority for future work.

## 5. Conclusions

In this prospective 6-month cohort, head-and-neck reconstruction was associated with broad improvements in physical and mental quality of life, alongside meaningful gains in body image, but recovery remained heterogeneous. Pedicled reconstruction was linked to substantially higher PEG dependence and worse swallowing at 6 months, while major complications strongly disrupted psychological recovery and increased rates of clinically significant anxiety and depression. Baseline swallowing function, radiotherapy exposure, and postoperative complications can help identify patients needing intensified multidisciplinary rehabilitation and psychosocial support. These findings are best interpreted as hypothesis-generating and support risk-adapted multidisciplinary follow-up after major head-and-neck reconstruction.

## Figures and Tables

**Table 1:** Baseline characteristics by reconstruction technique.

Variable	Total (*n* = 87)	Free Flap (*n* = 52)	Pedicled Flap (*n* = 35)	*p*-Value
Age, years (mean ± SD)	56.8 ± 11.2	55.1 ± 10.8	59.4 ± 11.6	0.086
Male sex, *n* (%)	61 (70.1)	34 (65.4)	27 (77.1)	0.349
BMI, kg/m^2^ (mean ± SD)	27.1 ± 4.4	26.8 ± 4.2	27.4 ± 4.6	0.539
Charlson Comorbidity Index (mean ± SD)	3.1 ± 1.4	2.9 ± 1.3	3.4 ± 1.6	0.097
Smoking status (current), *n* (%)	24 (27.6)	13 (25.0)	11 (31.4)	0.581
Advanced stage (III–IV), *n* (%)	55 (63.2)	38 (73.1)	17 (48.6)	0.036
Composite defect (bone + soft tissue), *n* (%)	23 (26.4)	19 (36.5)	4 (11.4)	0.018
Planned adjuvant radiotherapy, *n* (%)	60 (69.0)	40 (76.9)	20 (57.1)	0.086
History of anxiety/depression, *n* (%)	16 (18.4)	8 (15.4)	8 (22.9)	0.548
Primary site: oral/oropharynx, *n* (%)	49 (56.3)	29 (55.8)	20 (57.1)	0.934
T category (T1/T2/T3/T4), *n*	10/23/28/26	4/12/18/18	6/11/10/8	0.336
N category (N0/N1/N2/N3), *n*	38/23/23/3	20/14/16/2	18/9/7/1	0.616
M1 (distant metastasis), *n* (%)	0 (0.0)	0 (0.0)	0 (0.0)	—
Subsite [oral cavity/oropharynx/hypopharynx/larynx/other-composite], *n*	30/19/12/15/11	18/11/7/9/7	12/8/5/6/4	0.999

BMI, body mass index; SD, standard deviation. TNM categories follow the 8th-edition AJCC/UICC classification; T and N rows give counts per category (free-flap/pedicled-flap columns); cell entries separated by “/” correspond to the categories listed in the row label in the same order. *p*-values for T and subsite distributions are from Fisher’s exact test. In each row, the per-category counts sum to the corresponding group totals (free flap = 52, pedicled flap = 35, total = 87); the subsite distribution is consistent with the dichotomized oral-cavity/oropharynx total of 49 (30 + 19). All patients were M0 at presentation.

**Table 2:** Surgical course and functional outcomes at 6 months by reconstruction technique.

Outcome	Free Flap (*n* = 52)	Pedicled Flap (*n* = 35)	*p*-Value
Operative time, hours (mean ± SD)	7.4 ± 1.6	4.8 ± 1.4	<0.001
Length of stay, days (mean ± SD)	12.3 ± 4.6	10.7 ± 4.1	0.093
Any 30-day complication, *n* (%)	18 (34.6)	14 (40.0)	0.776
Major complication (Clavien–Dindo ≥ III), *n* (%)	8 (15.4)	4 (11.4)	0.835
Revision surgery ≤ 30 days, *n* (%)	9 (17.3)	3 (8.6)	0.4
Donor-site complication, *n* (%)	12 (23.1)	2 (5.7)	0.038
Tracheostomy dependence at 6 mo, *n* (%)	6 (11.5)	8 (22.9)	0.266
PEG dependence at 6 mo, *n* (%)	5 (9.6)	13 (37.1)	0.005
FOIS at 6 mo (1–7), mean ± SD	5.6 ± 1.2	4.7 ± 1.4	0.003
Speech intelligibility (0–100), mean ± SD	82.7 ± 10.6	78.4 ± 12.2	0.094

FOIS, Functional Oral Intake Scale; PEG, percutaneous endoscopic gastrostomy; SD, standard deviation.

**Table 3:** SF-36 domain scores at baseline and 6 months.

Domain	Baseline Mean ± SD	6 mo Mean ± SD	Δ Mean ± SD	*p*-Value	Cohen’s d
Physical Functioning	52.9 ± 13.4	59.7 ± 13.9	+6.8 ± 9.3	<0.001	0.7
Role Physical	48.6 ± 14.2	54.1 ± 14.6	+5.5 ± 10.6	<0.001	0.5
Bodily Pain	56.4 ± 15.1	61.1 ± 15.2	+4.7 ± 9.7	<0.001	0.5
General Health	50.7 ± 12.8	56.3 ± 13.1	+5.6 ± 9.0	<0.001	0.6
Vitality	48.9 ± 13.7	54.7 ± 14.0	+5.8 ± 10.1	<0.001	0.6
Social Functioning	54.3 ± 14.4	56.4 ± 14.9	+2.1 ± 11.7	0.098	0.2
Role Emotional	51.2 ± 15.0	56.8 ± 15.4	+5.6 ± 11.2	<0.001	0.5
Mental Health	50.4 ± 13.1	56.7 ± 13.6	+6.3 ± 9.4	<0.001	0.7

SF-36, 36-Item Short-Form Health Survey; SD, standard deviation; Δ, change from baseline to 6 months.

**Table 4:** WHOQOL-BREF domains and Body Image Scale (BIS) at baseline and 6 months.

Measure	Baseline Mean ± SD	6 mo Mean ± SD	Δ Mean ± SD	*p*-Value	Cohen’s d
WHOQOL Physical	52.7 ± 12.6	58.9 ± 12.7	+6.2 ± 8.3	<0.001	0.7
WHOQOL Psychological	54.8 ± 13.2	60.3 ± 13.5	+5.5 ± 8.9	<0.001	0.6
WHOQOL Social	56.1 ± 14.0	59.4 ± 14.6	+3.3 ± 9.8	0.002	0.3
WHOQOL Environmental	60.4 ± 13.5	63.1 ± 13.9	+2.7 ± 9.4	0.009	0.3
Body Image Scale (0–30; lower = better)	16.1 ± 5.8	13.2 ± 5.9	−2.9 ± 4.6	<0.001	−0.6

BIS, Body Image Scale; SD, standard deviation; WHOQOL-BREF, World Health Organization Quality of Life–BREF; Δ, change from baseline to 6 months.

**Table 5:** Psychological symptom trajectories by major complication status.

Scale	No Major Complication (*n* = 75) Baseline	No Major Complication 6 mo	Δ Mean ± SD	Major Complication (*n* = 12) Baseline	Major Complication 6 mo	Δ Mean ± SD	*p* (Δ Difference)
HADS-A	10.6 ± 3.7	8.0 ± 3.6	−2.6 ± 3.4	11.3 ± 3.8	11.1 ± 3.9	−0.2 ± 3.2	0.03
HADS-D	9.7 ± 3.5	7.4 ± 3.4	−2.3 ± 3.3	10.6 ± 3.7	11.0 ± 3.9	+0.4 ± 3.1	0.014
PHQ-9	11.1 ± 4.6	7.7 ± 4.4	−3.4 ± 3.8	12.8 ± 4.9	12.4 ± 5.0	−0.4 ± 3.9	0.026
GAD-7	9.4 ± 4.1	6.3 ± 3.9	−3.1 ± 3.6	10.2 ± 4.3	10.3 ± 4.4	+0.1 ± 3.5	0.01

GAD-7, Generalized Anxiety Disorder-7; HADS-A, Hospital Anxiety and Depression Scale–Anxiety; HADS-D, Hospital Anxiety and Depression Scale–Depression; PHQ-9, Patient Health Questionnaire-9; SD, standard deviation; Δ, change from baseline to 6 months.

**Table 6:** Clinically significant symptoms at 6 months.

Threshold	No Major Complication (*n* = 75)	Major Complication (*n* = 12)	*p*-Value
HADS-A ≥ 11, *n* (%)	19 (25.3)	7 (58.3)	0.037
HADS-D ≥ 11, *n* (%)	14 (18.7)	8 (66.7)	0.001
PHQ-9 ≥ 10, *n* (%)	18 (24.0)	7 (58.3)	0.034
GAD-7 ≥ 10, *n* (%)	15 (20.0)	7 (58.3)	0.009

GAD-7, Generalized Anxiety Disorder-7; HADS-A, Hospital Anxiety and Depression Scale–Anxiety; HADS-D, Hospital Anxiety and Depression Scale–Depression; PHQ-9, Patient Health Questionnaire-9.

**Table 7:** Correlations among 6-month change scores.

Variable 1 (Δ)	Variable 2 (Δ)	r	*p*-Value
BIS	SF-36 Mental Component Summary (MCS)	−0.52	<0.001
BIS	PHQ-9	0.49	<0.001
GAD-7	SF-36 MCS	−0.56	<0.001
FOIS	SF-36 Physical Component Summary (PCS)	0.41	<0.001
FOIS	PHQ-9	−0.33	0.002
WHOQOL Environmental	GAD-7	−0.21	0.051

BIS, Body Image Scale; FOIS, Functional Oral Intake Scale; GAD-7, Generalized Anxiety Disorder-7; MCS, Mental Component Summary; PCS, Physical Component Summary; PHQ-9, Patient Health Questionnaire-9; r, correlation coefficient; SF-36, 36-Item Short-Form Health Survey; WHOQOL, World Health Organization Quality of Life; Δ, change from baseline to 6 months.

**Table 8:** Multivariable logistic regression predicting PEG dependence at 6 months.

Predictor	aOR (95% CI)	*p*-Value
Pedicled flap (vs free flap)	4.6 (1.4–15.6)	0.0136
Baseline FOIS (per +1 point)	0.5 (0.3–0.8)	0.0077
Adjuvant radiotherapy (yes)	2.8 (1.1–7.2)	0.0393
Major complication (yes)	3.3 (1.1–9.8)	0.0351
Composite defect (yes) ‡	2.2 (0.8–6.0)	0.1387
Age (per +10 years) ‡	1.3 (0.9–2.1)	0.1874
Current smoking (yes) ‡	1.8 (0.8–4.3)	0.156
Advanced TNM stage (III–IV) †	1.5 (0.5–4.6)	0.49
Charlson Comorbidity Index (per +1 point) †	1.2 (0.8–1.7)	0.34

Model: multivariable logistic regression (Wald tests); odds ratios are adjusted (aOR); Outcome: PEG dependence at 6 months (yes/no); Model performance: Nagelkerke R^2^ = 0.43; AUC = 0.86; Hosmer–Lemeshow *p* = 0.612; aOR, adjusted odds ratio; AUC, area under the curve; CI, confidence interval; FOIS, Functional Oral Intake Scale; PEG, percutaneous endoscopic gastrostomy; R^2^, coefficient of determination. The primary model was obtained by backward elimination. † Advanced TNM stage and Charlson Comorbidity Index were added only in a pre-specified sensitivity model to control for tumor severity and comorbidity; neither was significant, and the pedicled-flap effect was essentially unchanged (aOR 4.2, 95% CI 1.2–14.8). Internal validation by 1000 bootstrap resamples gave an optimism-corrected AUC of 0.79 and a calibration slope of 0.84. TNM, tumor–node–metastasis. The primary model is the reduced four-predictor model (pedicled flap, baseline FOIS, adjuvant radiotherapy, and major complication; 18 events, 4.5 events per variable). ‡ Composite defect, age, and current smoking were pre-specified candidate covariates that were screened but did not survive backward elimination (all *p* > 0.10) and are shown only for transparency; they are not part of the reduced primary model. With four predictors retained, the optimism-corrected AUC of 0.79 (calibration slope 0.84) is the reported measure of discrimination, and the apparent AUC of 0.86 is shown only as the uncorrected value.

**Table 9:** Unsupervised “recovery phenotypes” based on multidimensional change.

Variable	Phenotype A: Functional and Emotional Responders (*n* = 34)	Phenotype B: Psychological/Body-Image Responders (*n* = 28)	Phenotype C: Global Slow Recovery (*n* = 25)	*p*-Value
ΔFOIS (mean ± SD)	+2.1 ± 1.1	+1.2 ± 1.3	+0.6 ± 1.0	<0.001
ΔSF-36 PCS (mean ± SD)	+8.2 ± 6.4	+5.1 ± 6.0	+2.1 ± 5.8	0.0012
ΔSF-36 MCS (mean ± SD)	+7.4 ± 6.1	+9.1 ± 6.6	+1.9 ± 6.2	0.0002
ΔPHQ-9 (mean ± SD)	−4.2 ± 3.1	−4.6 ± 3.4	−0.8 ± 3.6	<0.001
ΔGAD-7 (mean ± SD)	−3.8 ± 3.0	−4.2 ± 3.2	−0.6 ± 3.3	<0.001
ΔBIS (mean ± SD)	−3.8 ± 3.4	−4.6 ± 3.7	−0.7 ± 3.1	0.0002
PEG dependence at 6 mo, *n* (%)	2 (5.9)	6 (21.4)	10 (40.0)	0.006
Major complication, *n* (%)	3 (8.8)	2 (7.1)	7 (28.0)	0.05
Pedicled flap, *n* (%)	9 (26.5)	12 (42.9)	14 (56.0)	0.0691

Method: Gaussian-mixture (latent profile–style) clustering on standardized 6-month change scores: ΔFOIS, ΔSF-36 MCS, ΔPHQ-9, ΔBIS. Group comparisons: one-way ANOVA (continuous) + χ^2^ (categorical); BIS, Body Image Scale; FOIS, Functional Oral Intake Scale; GAD-7, Generalized Anxiety Disorder-7; MCS, Mental Component Summary; PCS, Physical Component Summary; PHQ-9, Patient Health Questionnaire-9; SF-36, 36-Item Short-Form Health Survey; Δ, change from baseline to 6 months.

**Table 10:** Parallel mediation analysis: Is reconstruction technique associated with mental QoL improvement through swallowing and body image?

Path	Coefficient (b) ± SE	*p*-Value
a_1_: Pedicled → ΔFOIS	−0.8 ± 0.3	0.0038
a_2_: Pedicled → ΔBIS	+1.4 ± 0.7	0.0412
b_1_: ΔFOIS → ΔMCS	+1.6 ± 0.5	0.0027
b_2_: ΔBIS → ΔMCS	−0.5 ± 0.2	0.0091
c (total): Pedicled → ΔMCS	−4.1 ± 1.6	0.0104
c′ (direct): Pedicled → ΔMCS	−2.1 ± 1.3	0.1117

Model: Pedicled (1) vs. Free (0) → ΔSF-36 MCS; mediators (parallel): ΔFOIS and ΔBIS; Method: bootstrapped mediation (5000 resamples), adjusted for age, adjuvant radiotherapy, major complication, baseline MCS, and baseline FOIS; BIS, Body Image Scale; FOIS, Functional Oral Intake Scale; MCS, Mental Component Summary; SE, standard error; SF-36, 36-Item Short-Form Health Survey; Δ, change from baseline to 6 months.

## Data Availability

The fully anonymized patient-level dataset underlying all analyses has been provided to the editorial office for peer review and editorial verification. Because the data contain potentially identifying clinical information from a single-center cohort, public posting is restricted for privacy and ethical reasons; de-identified data are otherwise available from the corresponding author on reasonable request, subject to a data-use agreement and the approvals of the institutional ethics committee.

## Appendix A. STROBE Item-Location Checklist

The STROBE reporting checklist is embedded below within the main manuscript. Page/section locations refer to the present revised version (Table A1).

**Table A1:** STROBE Checklist.

Section	STROBE Item	Reported Location in Manuscript
Title and abstract	1(a) Indicate the study’s design with a commonly used term in the title or the abstract.	Title; Abstract
Title and abstract	1(b) Provide in the abstract an informative and balanced summary of what was done and what was found.	Abstract
Introduction	2 Explain the scientific background and rationale for the investigation being reported.	Introduction, paragraphs 1–6
Introduction	3 State specific objectives, including any prespecified hypotheses.	End of Introduction
Methods	4 Present key elements of study design early in the paper.	Section 2.1
Methods	5 Describe the setting, locations, and relevant dates, including periods of recruitment, exposure, follow-up, and data collection.	Section 2.1
Methods	6(a) Give the eligibility criteria, and the sources and methods of selection of participants. Describe methods of follow-up.	Section 2.2; Figure 1
Methods	6(b) For matched studies, give matching criteria and number of exposed and unexposed.	Not applicable
Methods	7 Clearly define all outcomes, exposures, predictors, potential confounders, and effect modifiers. Give diagnostic criteria, if applicable.	Section 2.2, Section 2.3 and Section 2.4
Methods	8 For each variable of interest, give sources of data and details of methods of assessment. Describe comparability of assessment methods if there is more than one group.	Section 2.2 and Section 2.3
Methods	9 Describe any efforts to address potential sources of bias.	Section 2.1, Section 2.4 and Section 4.2
Methods	10 Explain how the study size was arrived at.	Section 2.4
Methods	11 Explain how quantitative variables were handled in the analyses. If applicable, describe which groupings were chosen and why.	Section 2.4
Methods	12(a) Describe all statistical methods, including those used to control for confounding.	Section 2.4
Methods	12(b) Describe any methods used to examine subgroups and interactions.	Section 2.4; Table 5, Table 8, Table 9 and Table 10
Methods	12(c) Explain how missing data were addressed.	Figure 1; Results opening paragraph
Methods	12(d) If applicable, explain how loss to follow-up was addressed.	Figure 1; Results opening paragraph
Methods	12(e) Describe any sensitivity analyses.	Exploratory analyses described in Section 2.4; Table 8, Table 9 and Table 10
Results	13(a) Report numbers of individuals at each stage of study.	Figure 1; Results opening paragraph
Results	13(b) Give reasons for non-participation at each stage.	Figure 1; Results opening paragraph
Results	13(c) Consider use of a flow diagram.	Figure 1
Results	14(a) Give characteristics of study participants and information on exposures and potential confounders.	Table 1
Results	14(b) Indicate number of participants with missing data for each variable of interest.	Results opening paragraph; Figure 1
Results	14(c) Summarize follow-up time.	Section 2.1; Results opening paragraph
Results	15 Report numbers of outcome events or summary measures over time.	Table 2, Table 3, Table 4, Table 5, Table 6, Table 7, Table 8, Table 9 and Table 10
Results	16(a) Give unadjusted estimates and, if applicable, confounder-adjusted estimates and their precision.	Table 2, Table 3, Table 4, Table 5, Table 6, Table 7, Table 8, Table 9 and Table 10
Results	16(b) Report category boundaries when continuous variables were categorized.	Table 5 and Table 6; Section 2.3
Results	16(c) If relevant, consider translating estimates of relative risk into absolute risk for a meaningful time period.	Table 8; Figure 2 narrative
Results	17 Report other analyses done, for example, analyses of subgroups and interactions, and sensitivity analyses.	Table 5, Table 7, Table 8, Table 9 and Table 10
Discussion	18 Summarize key results with reference to study objectives.	Section 4.1
Discussion	19 Discuss limitations of the study, taking into account sources of potential bias or imprecision.	Section 4.2
Discussion	20 Give a cautious overall interpretation of results considering objectives, limitations, multiplicity of analyses, results from similar studies, and other relevant evidence.	Section 4.1 and Section 4.2
Discussion	21 Discuss the generalizability of the study results.	Section 4.2; Section 5 (Conclusions)
Other information	22 Give the source of funding and the role of the funders.	Funding statement
